# Parental perception of the application of silver diamine fluoride (SDF) for dental caries treatment among Iraqi school children: a cross-sectional study

**DOI:** 10.1038/s41405-025-00307-x

**Published:** 2025-02-13

**Authors:** Ammar Albujeer, Hadi Ghasemi, Mahshid Namdari, Abbas Taher, Alya Almahafdha, Mohammad H. Khoshnevisan

**Affiliations:** 1 Community Oral Health Department, School of Dentistry, Shahid Beheshti University of Medical Sciences, Tehran, Iran; 2 Health Research Unit, Nab’a Al-Hayat Foundation for Medical Sciences and Health Care, Najaf, Iraq; 3 Oral & Maxillofacial Surgery Department, School of Dentistry, Islamic University, Najaf, Iraq; 4 Dental Research Center, Research Institute of Dental Sciences, School of Dentistry, Shahid Beheshti University of Medical Sciences, Tehran, Iran

**Keywords:** Minimal intervention dentistry, Paediatric dentistry

## Abstract

**Objectives:**

The present study aimed to assess parental acceptance of silver diamine fluoride (SDF) treatment for dental caries in children in Najaf city, Iraq.

**Methods:**

A cross-sectional design was used, involving 670 parents of children aged 6–7 years from primary schools in Najaf city, Iraq, during the academic year 2023–2024. The participants were selected via a multistage random sampling method. A structured questionnaire was administered to gather the participants’ views on tooth staining caused by SDF application to cavitated teeth. Statistical procedures included descriptive analysis, chi-square tests, and ordinal logistic regression.

**Results:**

In total, 670 parents were recruited for this study. The mean age of the participants was 34.47 ± 8.2 Approximately half of the respondents were male and aged 31–50 years, and approximately three-fourths of the parents reported having a low education level and were in the low-income category. Parental acceptance of SDF treatment was greater for posterior teeth, with 51.2% agreeing, and 24.2% strongly agree. For anterior teeth, acceptance was lower, with only 23.3% agreeing and 10.7% strongly agreeing. Parental age (*p* = 0.008), education level (*p* < 0.001), and income (*p* = 0.003) were significantly associated with acceptance of SDF treatment for posterior teeth. However, for anterior teeth, parental education (*p* < 0.001) and income (*p* = 0.029) were significantly associated with acceptance of SDF treatment.

**Conclusion:**

Parents showed high acceptance of SDF treatment, particularly for posterior teeth, although concerns about aesthetics affected their views of anterior teeth. Improving parents’ awareness and addressing their apprehensions could increase the adoption of this evidence-based caries management approach for children.

## Introduction

Dental caries is a highly widespread chronic disease that affects children globally, regardless of their socioeconomic status [1]. Despite progress in dental care and preventive initiatives, this common disease still poses important public health challenges [2]. Dental caries susceptibility is affected by various factors, such as socioeconomic level, dietary habits, educational achievement, and demographic variables [3]. The combination of these components increases the complexity of effectively controlling and preventing dental caries, and continuous research and investigations of inventive treatment options are needed [4].

The treatment of dental caries typically involves two main approaches: restorative and preventative [5]. Restorative treatment, which involves invasive procedures such as drilling and filling, can be expensive and may not be easily available to all people, especially in resource-limited situations [6]. Preventive techniques are intended mainly to stop the initiation and/or progression of tooth decay by promoting remineralization. These attractive techniques have been found to be practical options [7]. Among these options, SDF has emerged as a promising solution, especially when a cavity is formed and conventional treatments are not accessible or feasible [8].

SDF is a straightforward topical substance that has proven effective in stopping dental decay by creating a protective barrier that blocks the channels in exposed dentin [9]. The silver ingredient in SDF hinders the development of biofilms by oral microorganisms, therefore preventing the progression of decay [10]. SDF has been endorsed by the American Dental Association and the American Academy of Pediatric Dentistry as a safe, feasible, cost-effective, and minimally intrusive treatment for active dental caries [11].

SDF treatment has demonstrated efficacy in controlling dental caries in children [12]. Numerous studies have substantiated its effectiveness in arresting caries progression. For example, a systematic review by Zaffarano et al. revealed that the application of SDF to active cavitated caries lesions in primary molars effectively halts the progression of dental caries, particularly with biannual application [8]. Similarly, another recent systematic review by Muntean et al. (2024) demonstrated that SDF is a convenient, accessible, and efficient non-invasive method with no need for anaesthesia injection for preventing or stopping caries in both primary and permanent teeth, emphasizing the importance of regular monitoring during its application [13]. Despite these benefits, the use of SDF remains limited. One significant barrier to its widespread adoption is the reluctance of parents to accept SDF as a treatment option for their children [14].

The utilization of SDF treatment in paediatric dentistry is influenced by several factors. Aesthetic concerns are a primary issue, as SDF can cause dark staining of treated teeth, which may be less acceptable to parents, especially for anterior teeth [15]. Socioeconomic factors, including income and access to dental care, affect the adoption of SDF, as those with higher education and income levels are more likely to be informed about and accept preventive treatments [16]. Additionally, cultural beliefs and norms about dental aesthetics and treatment approaches can influence parental decisions [17]. The availability and recommendation of SDF by dental professionals can further impact its use. Additionally, clinicians’ attitudes and communication skills are critical in addressing parents’ concerns and promoting acceptance of this treatment option [18].

Studies suggest that the level of parental acceptability of SDF differs depending on cultural context and individuals’ opinions of the advantages of the treatment. For example, a study carried out in China revealed that parents showed little concern about staining after SDF was applied [19]. Studies conducted in the United States and the United Kingdom reported different levels of acceptance, with a preference for using SDF on back teeth rather than front teeth for aesthetic reasons [20, 21]. A study conducted in Iran and Tajikistan reported parental acceptance of the use of SDF for treating primary teeth. However, for permanent teeth, treating posterior teeth was acceptable [22].

The selection of Iraq as the study location is especially relevant because of the country’s distinct socioeconomic environment and the difficulties encountered in obtaining dental healthcare. The healthcare system in Iraq has been greatly affected by long-lasting conflict and economic instability. As a result, there is a need to investigate cost-effective and minimally invasive treatments such as SDF. Furthermore, the cultural perspectives on dental aesthetics and treatment methods in Iraq may vary from those seen in other areas, requiring a customized approach to comprehending and resolving parental concerns.

Despite the growing body of literature on SDF, there is a notable lack of research focusing on parental attitudes towards SDF treatment in Iraq. The socioeconomic context, cultural attitudes, and healthcare infrastructure in Iraq present unique challenges and opportunities for implementing SDF as a caries management strategy. Understanding parental views in this context is crucial for developing effective dental health policies and programs tailored to the needs and preferences of the Iraqi population. This study aimed to investigate parental views on the application of SDF for dental caries treatment among Iraqi school children.

## Methodology

### Target population and sampling

The target population for this study consisted of parents of children aged 6–7 years who attended primary schools in Najaf city. The required sample size was calculated via the Raosoft sample size calculator, with Najaf’s population size estimated at approximately 1221248 people [23] and 251605 cases of the 6–7-year-old target population. The confidence interval was set at 95%, the response distribution was 50%, the margin of error was set at 4%, and the calculated sample size was 600. Due to probable nonresponding participants, approximately 10% was added to the sample size, which resulted in a total of 670 parents for this study.

The inclusion criteria for the study were that, both parents and children be of Iraqi nationality and 6–7-year-old healthy children had at least one asymptomatic anterior/posterior tooth with caries. The exclusion criteria were children who were free of any dental caries, children with medical condition or allergy to silver products. Also children having pain or infection in their decayed teeth or parents who did not wish to participate in the study.

### Study design

This study utilized a cross-sectional design. In this descriptive study, data were collected from a sample of recruited parents over a short period of time (3 months, October to December 2023) via a structured questionnaire (25). The study was ethically approved by the institutional review board of Shahid Beheshti University of Medical Sciences and Health Services, with reference number IR.SBMU.DRC.REC.1402.116. Permissions were also obtained from the General Directorate of Education of Najaf and the principals of the selected schools, ensuring that all institutional requirements were met. Informed consent was obtained from each participating parent, and the purpose of the study, procedures, potential risks, and benefits were thoroughly explained. The participants were assured that their involvement was voluntary and that they could withdraw from the study at any time without any consequences. All the data collected were kept confidential and anonymized to protect the participants’ confidentiality. Additionally, necessary measures were taken to protect participants’ privacy.

### Sample recruitment

Sample selection was conducted in two stages. In the first stage, districts and schools (clusters) were selected via the stratified-cluster sampling method. Al-Najaf City has 235 primary schools. Ten primary schools were randomly selected via the random number method [24]. In the second stage, students were stratified and selected by age and/or grade 1, using disproportionate stratified sampling. All the schools participating in the study had several grade one classrooms, each consisting of about 40 students. At this point, all Grade one students were included in the study to undergo oral examination. For this study, we needed students with cavitated dental caries only.

### Data collection

A team of 4 dental students (in the final year) from the Islamic University of Najaf, with the principal investigator, conducted clinical oral examinations on children from selected schools. A pilot test was performed to assess inter- and intraexaminer reliability in a sample of 67 children (10% of the total sample), who were selected from a different school that was not among the previously selected primary schools. The WHO protocol (2013) was used for the calibration of the examiners for dental caries diagnosis. The same children were re-examined after two weeks. The interexaminer and intraexaminer reliability and kappa values were 0.95 and 0.91, respectively, indicating good reliability between the examiners. The children involved in this reliability test were not included in the main study. Children identified with dental caries in the pilot study were given a questionnaire and a consent form to take home, which was signed by their parents and returned on the following days for proper guidance and referral. After the oral examination of 720 children attending 18 classrooms, a total of 670 children who submitted signed consent forms were included in the study.

Throughout the process, the principal investigator was available to address any questions or concerns from both students and parents, ensuring clear communication and a thorough understanding of the study’s objectives. Data collection was conducted via a structured questionnaire in the Arabic language. To evaluate the questionnaire’s reliability through the test‒retest method, 10% of the nonparticipating sample was given the questionnaire twice under the same conditions, with a 10-day interval between the 2 administrations. The correlation coefficient (r) of 0.89 between the two time points indicates acceptable reliability for the questionnaire.

The questionnaire was composed of three sections. The demographic, socioeconomic and parental views (each section with 2 questions) [25]. The following information was collected from each participant: age, sex, level of education, income, and their opinions on the staining shown in the photographs after SDF application to the anterior and posterior decayed teeth.

To evaluate the aesthetic impact of SDF application before treatment, parents were shown a series of photographs taken before and after the treatment for comparison. These images highlighted the condition of both anterior and posterior teeth, showcasing the presence of carious enamel and dentin prior to and following the SDF application. Parents’ views on the aesthetics and their acceptance of the staining caused by SDF were collected and summarized based on their feedback.

To ensure that parents were well informed before providing their opinions, the introduction to the questionnaire provided detailed information about the advantages, disadvantages, indications, and contraindications of SDF. Parental acceptance of SDF treatment was measured via a 5-point Likert scale ranging from strongly disagree to strongly agree, with scores ranging from 0 to 4.

### Statistical analysis

The collected data were tabulated and analysed via IBM SPSS Statistics software version 26. The descriptive data are expressed as the means, standard deviations (SDs), frequencies, and percentages (%). The chi-square test was used to compare the groups to determine the relationships between the dependent and independent variables. Ordinal regression analysis was conducted to determine factors associated with the dependent variable (parental acceptance of SDF treatment), and statistical significance was established at a *p* value of less than 0.05.

## Results

A total of 670 parents participated in this study. The mean age was 34.47 ± 8.2 years. Table 1 presents the descriptive characteristics of the study population. Almost half of the respondents were male and were aged 31–50 years, and approximately three-fourths of the parents reported having a low education level and belonging to the category of low income. A single child was reported by less than one-fifth of the families.

**Table 1:** Background characteristics of the Iraqi parents who participated in the study (*n* = 670)

Variables	Frequency (*n*)	Percentage (%)
**Age of Parents**		
17–30	225	33.6
31–50	399	59.6
>51	46	6.8
**Gender**		
Male	343	51.2
Female	327	48.8
**Education Level**		
Educated (Higher Education + Basic Education)	603 (82 + 521)	90.0 (12.1 + 77.9)
Illiterate	67	10.0
**Income**		
Low	388	57.9
High	282	42.1
**Number of Children**		
1–4	570	85.0
5–9	100	15.0

**Fig. 1:**
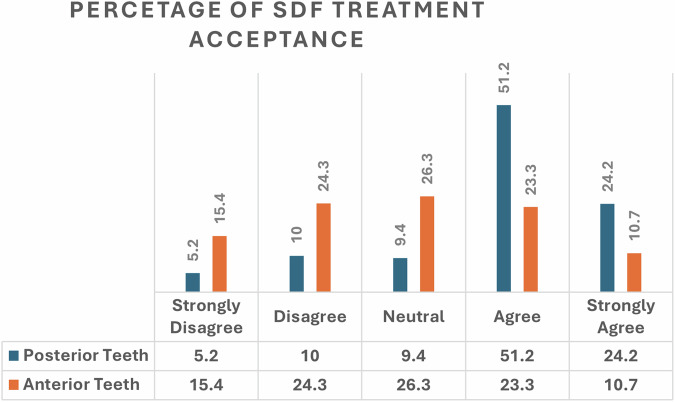
Acceptance levels of Iraqi parents regarding SDF treatment for their children’s teeth. This figure illustrates the percentage distribution of parental acceptance levels for silver diamine fluoride (SDF) treatment. The data are categorized into different acceptance levels, reflecting parental perspectives on this dental intervention.

Figure 1 shows differences in parental acceptance levels of SDF treatment for anterior and posterior teeth. For posterior teeth, approximately three-fourths of the parents (75.4%) agreed on SDF treatment for their children. For anterior teeth, the acceptance level was reported by approximately one-third (34%) of the parents. The overall rate of parental acceptance of SDF application was 86.1%.

Table 2 shows the relationships between parental demographic characteristics and acceptance of SDF treatment for posterior and anterior teeth. For posterior teeth, more acceptance was observed, including parents who were younger (*p* = 0.008), educated (*p* < 0.001), and low income (*p* = 0.003). For the anterior teeth, more acceptance was reported for educated (*p* < 0.001) parents and those with lower incomes (*p* = 0.029).

**Table 2:** Distribution (%) of Iraqi parents’ (*n* = 670) level of agreement (agree + strongly agree) regarding treatment of their children’s teeth with SDF separately for the anterior and posterior teeth.

Variables	SDF Treatment Acceptance for Posterior Teeth (%)	*p* value^*^	SDF Treatment Acceptance for Anterior Teeth (%)	*p* value^*^
**Age**				
17–30	25.82	**0.008**^**a**^	10.29	0.110
31–50	44.47		20.89	
>51	5.07		2.83	
**Gender**				
Male	37.46	0.448	18.20	0.335
Female	37.91		15.82	
**Education Level**				
Educated	68.50	<**0.001**^**a**^	30.89	<**0.001**^**a**^
Illiterate	6.86		3.13	
**Income**				
Low	44.02	**0.003**^**a**^	21.79	**0.029**^**a**^
High	31.34		12.23	
**Number of Children**				
1–4	428	0.732	29.25	0.256
5–9	77		4.77	

*Statistical evaluation by the chi-square test.

Table 3 shows the factors associated with parents’ acceptance of SDF treatment as analysed by two ordinal logistic regression models while controlling for age, sex, level of education and number of children. For posterior teeth, fathers were more likely to agree than mothers were (β = 0.331, 95% CI = 0.626–0.036), and educated parents (β = 0.647, 95% CI = 0.097–1.196) were more likely to accept SDF treatment. For the anterior teeth, no factors had a significant effect on parents’ acceptance of SDF treatment.

**Table 3:** Factors determining Iraqi parents’ acceptance of SDF treatment for their children as assessed by two ordinal logistic regression models separately for the posterior and anterior teeth.

Variables	B	SE	Wald	*p* value	95% CI	
					Lower	Upper
**For Posterior Teeth**						
Age	−0.025	0.017	2.115	0.146	−0.058	0.009
Gender	−0.331	0.150	4.825	**0.028**^a^	0.626	0.036
Education Level	0.647	0.280	5.326	**0.021**^a^	0.097	1.196
Income	−0.376	0.362	1.082	0.298	−1.085	0.333
Number of Children	−0.312	1.942	0.026	0.872	−4.119	3.495
**For Anterior Teeth**						
Age	0.009	0.016	0.330	0.566	−0.022	0.040
Gender	0.059	0.141	1.76	0.675	−0.218	0.336
Education Level	−0.222	0.263	0.713	0.399	−0.736	0.293
Income	0.450	0.344	1.710	0.191	−0.224	1.124
Number of Children	0.774	1.851	0.175	0.676	−2.854	4.402

^a^Significant *p* value < 0.05; *β* beta coefficient, *SE* standard error, *Wald* Wald statistic, *CI* confidence interval.

## Discussion

The aim of the present study was to assess parental acceptance of silver diamine fluoride (SDF) treatment for children in Najaf city, Iraq. The decision to focus on school children aged 6–7 years was based on several considerations. First, this age group is easily accessible through primary schools, allowing for effective preventive activities through parents’ and teachers’ collaboration. Additionally, children in this age group are at the verge of permanent teeth eruption. This developmental stage presents an important opportunity for early intervention, as silver diamine fluoride (SDF) can help protect developing permanent teeth from caries that may result from the progression of decay in primary teeth. These findings underscore the distinct differences in parental acceptance levels of SDF treatment for anterior versus posterior teeth. These differences were driven primarily by aesthetic concerns, a factor that plays a pivotal role in decision-making within paediatric dentistry. The study revealed that while parents were generally more accepting of SDF treatment for posterior teeth, the acceptance rates decreased significantly for anterior teeth, where the potential for visible staining became a major concern.

Aesthetic considerations emerged as a critical determinant in parental acceptance of SDF treatment, particularly for anterior teeth. This finding is consistent with the literature, including studies by Crystal et al. [15] and Cappiello et al. [26], which similarly reported greater acceptance of SDF treatment for posterior teeth. These studies highlighted that, the reduced visibility of posterior teeth makes the aesthetic impact of SDF’s dark staining less concerning for parents. In contrast, anterior teeth, which are more visible, evoke stronger aesthetic concerns, leading to lower acceptance rates. The lower acceptance of SDF for anterior teeth, as revealed by this study, aligns with prior research [27, 28]. Parents’ reluctance to use SDF on anterior teeth, despite its proven efficacy in arresting dental caries, points to a broader issue within paediatric dentistry. This challenge is not unique to SDF but is a recurring theme in paediatric dental care, where the appearance of a child’s smile can significantly influence parental decisions.

The findings of this study consistently showed that parental acceptance of SDF treatment was greater for posterior teeth than for anterior teeth. Both the chi-square analysis and ordinal logistic regression revealed that, for posterior teeth, parental education, income, and sex were significant predictors of acceptance. The findings that higher levels of education and income are associated with greater acceptance of SDF treatment are also consistent with previous studies [25, 29–32]. Parents with higher education levels and greater financial resources are likely better informed about the benefits of preventive dental care, including SDF [32]. They probably had better access to dental health services and were more likely to seek out information about various treatment options, leading to more positive reception of SDF [33]. Parents from higher SES backgrounds often have the means to consult with multiple healthcare providers, seek second opinions, and access a wider range of treatment options [34]. This contrasts with parents from lower SES backgrounds, who may rely more heavily on the recommendations of a single provider and may not have the same level of access to information about treatments such as SDF [35].

The study also highlights sex differences in the acceptance of SDF treatment, particularly for posterior teeth. Compared with male parents, female parents were found to be less likely to accept SDF treatment. Similar findings were reported in previous studies [20, 25]. This finding was intriguing and suggests that mothers, who often serve as the primary decision-makers in matters related to their children’s health, may place greater emphasis on the aesthetic aspects of dental treatments [25]. This gender difference could be attributed to societal expectations and roles. In many cultures, mothers are expected to manage their family’s health and well-being, including making decisions about their children’s medical and dental care [36]. This responsibility might make mothers more attuned to the potential social implications of visible dental treatments, such as the staining caused by SDF. Moreover, mothers may be more sensitive to how their children’s appearance affects their social interactions and self-esteem, particularly in cultures where appearance plays a significant role in social recognition and success [37]. This could explain why the female parents in this study were less likely to accept SDF treatment, especially for teeth that were visible when a child talked or smiled. The findings of this study revealed no significant association between the number of children and parental acceptance of SDF treatment. This lack of association suggests that the decision to accept or reject SDF treatment was likely influenced more by factors such as aesthetic concerns, education, and income than by the number of children in the family. It may be that parents, regardless of how many children they had, prioritized the individual aesthetic outcomes and long-term dental health of each child when making treatment decisions.

The consistent finding that aesthetic concerns dominate the decision-making process for anterior teeth suggests that clinicians need to adopt a more nuanced approach when recommending SDF treatment. While the efficacy of SDF in arresting dental caries is well established, its aesthetic drawbacks must be addressed transparently with parents [17]. One potential strategy to improve acceptance rates is to combine SDF with other cosmetic treatments, such as the application of tooth-colored restorative materials [38]. This approach could mitigate the aesthetic concerns associated with SDF while still providing the caries-preventive benefits of treatment. By offering a comprehensive treatment plan that addresses both the health and aesthetic needs of the patient, clinicians can increase parental acceptance and satisfaction [31].

Additionally, clinicians should prioritize effective communication with parents, particularly when discussing the trade-offs between aesthetic outcomes and the health benefits of SDF [17]. This communication should be tailored to the individual concerns and cultural contexts of each family. For example, in cultures where aesthetic concerns are particularly strong, clinicians might spend more time discussing alternative treatments or the possibility of combining SDF with cosmetic procedures [39]. Educational interventions by care providers are also crucial [40]. By educating parents about the long-term benefits of SDF and its role in preventing more invasive procedures, such as extractions or fillings, clinicians can help parents understand the importance of preventive care [32]. These educational efforts should be culturally sensitive and consider the specific concerns that different demographic groups might have. Nevertheless, the findings of this study will contribute to a broader understanding of how SDF can be integrated into dental care practices in diverse settings, ultimately improving oral health outcomes for children.

### Limitations and future directions

The study was conducted in a specific geographic area, and the findings may not be generalizable to other populations with different cultural, social, or economic contexts. Additionally, the study relied on self-reported data from parents, which may be subject to bias. Future research should explore the acceptance of SDF in a broader range of populations, including those in different cultural settings and socioeconomic backgrounds. Longitudinal studies that track changes in parental attitudes over time would also be valuable, as they could provide insights into how acceptance of SDF evolves as parents gain more experience with the treatment or as new information becomes available. Further research is needed to develop and test new formulations of SDF that minimize its aesthetic impact while retaining its efficacy. Exploring the development of stainless SDF and/or the use of adjunctive treatments to reduce staining or improve the cosmetic outcomes of SDF application could also help increase its acceptance. The DMFT and dmft indices were assessed but not reported in this paper because assessing parental perception was the main goal of this study. These results will be reported separately.

## Conclusion

While Iraqi parents generally favour SDF treatment for posterior teeth, significant concerns persist for anterior teeth due to aesthetic considerations. To improve the acceptance and utilization of SDF, it is crucial to increase parental awareness and address apprehensions regarding its use. As an evidence-based approach for managing caries in children, SDF offers a viable solution to paediatric dental care. By effectively communicating the benefits and addressing the cosmetic concerns associated with SDF, dental professionals can foster greater parental acceptance. This, in turn, can lead to broader uptake of this treatment, ensuring that more children who require caries management receive the necessary care.

## Data Availability

The datasets generated and/or analyzed during the current study are available from the corresponding author on reasonable request.
